# Supplementary material for: Controlled Attenuation Parameter Is Associated with a Distinct Systemic Inflammatory Milieu after Clearance of HCV Infection
Source: Biomedicines. 2023 May 25;11(6):1529. doi: 10.3390/biomedicines11061529 (PMC10295384; doi:10.3390/biomedicines11061529)
Supplement: Supplementary file 1 [file biomedicines-11-01529-s001.zip › biomedicines-2372008-supplementary.pdf]

Supplementary data

Supplementary Figure S1

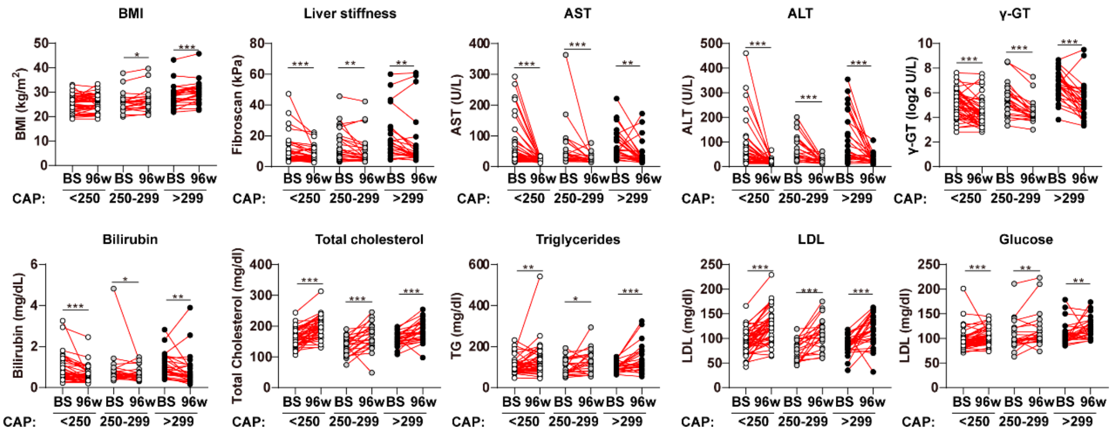

Supplementary figure S1. The summary of clinical parameters of these patients at baseline and 96w. Patients were classified into three groups according to their steatosis status at 96w: CAP < 250 dB/m (n = 44), CAP 250-299 dB/m (n = 23), CAP >299 dB/m (n = 27). Clinical parameters were summarized from baseline to 96w. Statistical analysis was performed via Wilcoxon matched-pairs signed rank test for two paired groups. **P* < 0.05, ***P* < 0.01, ****P* < 0.001. Abbreviations: BS, baseline; BMI, body mass index; AST, aspirate aminotransferase; ALT, alanine aminotransferase; γ-GT, gamma-glutamyl transferase; SIM, soluble inflammatory mediator; LDL, low-density lipoprotein.

Supplementary Figure S2

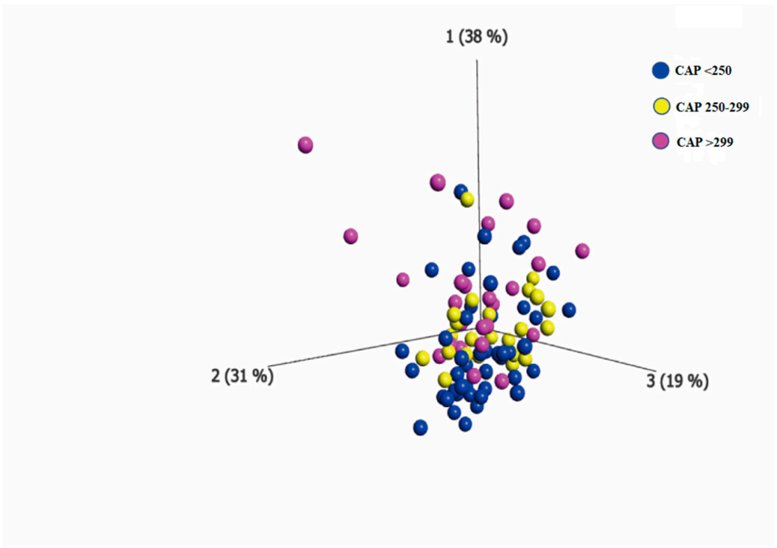

Supplementary figure S2. Principal component analysis (PCA) showed a distinct clustering of patients with different severity of steatosis. The PCA plot was calculated using 96w values of all 72 SIMs and shows how patients cluster together based on their steatosis status at 96w. Principal component analysis (PCA) and heatmap was performed using Qlucore Omics Explorer v3.6 (Qlucore, Lund, Sweden). Multiple group comparison was used in PCA to compare patients within different times points. For analysis, values were set to P values of .05 and a Q value of < 0.2.

Supplementary Figure S3

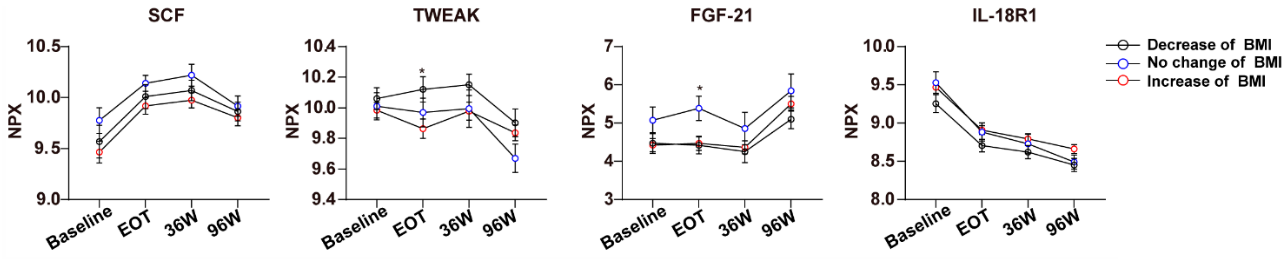

Supplementary Figure S3. The kinetics of SCF, TWEAK, FGF-21 and IL-18R1 among different change pattern of BMI. Patients were classified into three groups according to the change pattern of BMI from baseline to 96w: decrease of BMI (n = 24), no change of BMI (n = 17) and increase of BMI (n = 52). The kinetics of the four markers was shown. Statistical analysis was performed via ANOVA with Kruskal-Wallis test followed by Dunn's multiple comparison test. *P < 0.05, Median and standard errors of mean are presented.
